# (*E*)-2-[3-(Trifluoro­meth­yl)phenyl­imino­meth­yl]benzene-1,4-diol

**DOI:** 10.1107/S1600536809041610

**Published:** 2009-10-17

**Authors:** Zarife Sibel Şahin, Sümeyye Gümüş, Mustafa Macit, Şamil Işık

**Affiliations:** a Department of Physics, Faculty of Arts and Sciences, Ondokuz Mayıs University, Kurupelit, TR-55139 Samsun, Turkey; b Department of Chemistry, Faculty of Arts and Sciences, Ondokuz Mayıs University, TR-55139 Samsun, Turkey

## Abstract

In the title compound, C_14_H_10_F_3_NO_2_, the two benzene rings are oriented at a dihedral angle of 31.94 (14)°. An intra­molecular O—H⋯N hydrogen bond helps to stabilize the mol­ecular structure. In the crystal, inter­molecular O—H⋯O hydrogen bonding links the mol­ecules, forming chains running along the crystallographic *a* axis. The F atoms of the trifluoro­methyl group are disordered over two positions with refined site occupancies of 0.488 (5) and 0.512 (5).

## Related literature

For the biological properties of Schiff bases, see: Lozier *et al.* (1975 ▶). For Schiff base tautomerism, see: Şahin *et al.* (2005 ▶); Hadjoudis *et al.* (1987 ▶). For the structure of a similar compound, see: Temel *et al.* (2007 ▶). For classification of hydrogen-bonding patterns, see: Bernstein *et al.* (1995 ▶). For related structural studies of Schiff bases, see: (Gül *et al.*, 2007 ▶; Şahin *et al.*, 2009*a* ▶,*b* ▶,*c* ▶).
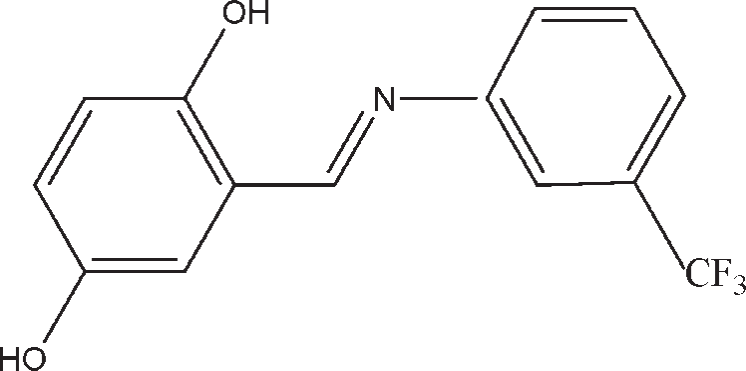


## Experimental

### 

#### Crystal data

C_14_H_10_F_3_NO_2_
*M* *_r_* = 281.23
Triclinic, 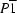

*a* = 7.1019 (8) Å
*b* = 8.5910 (8) Å
*c* = 11.0412 (11) Å
α = 73.862 (8)°
β = 74.133 (7)°
γ = 87.431 (8)°
*V* = 622.10 (11) Å^3^
*Z* = 2
Mo *K*α radiation
μ = 0.13 mm^−1^
*T* = 296 K
0.49 × 0.32 × 0.02 mm

#### Data collection

Stoe IPDS II diffractometer
Absorption correction: multi-scan (*X-RED32*; Stoe & Cie, 2002 ▶) *T*_min_ = 0.934, *T*_max_ = 0.995
6675 measured reflections
2548 independent reflections
1490 reflections with *I* > 2σ(*I*)
*R*_int_ = 0.073

#### Refinement

*R*[*F* ^2^ > 2σ(*F* ^2^)] = 0.100
*wR*(*F* ^2^) = 0.293
*S* = 1.07
2548 reflections
183 parameters
H atoms treated by a mixture of independent and constrained refinement
Δρ_max_ = 0.62 e Å^−3^
Δρ_min_ = −0.56 e Å^−3^

### 

Data collection: *X-AREA* (Stoe & Cie, 2002 ▶); cell refinement: *X-AREA*; data reduction: *X-RED32* (Stoe & Cie, 2002 ▶); program(s) used to solve structure: *SHELXS97* (Sheldrick, 2008 ▶); program(s) used to refine structure: *SHELXL97* (Sheldrick, 2008 ▶); molecular graphics: *ORTEP-3 for Windows* (Farrugia, 1997 ▶); software used to prepare material for publication: *WinGX* (Farrugia, 1999 ▶).

## Supplementary Material

Crystal structure: contains datablocks I, global. DOI: 10.1107/S1600536809041610/xu2631sup1.cif

Structure factors: contains datablocks I. DOI: 10.1107/S1600536809041610/xu2631Isup2.hkl

Additional supplementary materials:  crystallographic information; 3D view; checkCIF report

## Figures and Tables

**Table 1:** Hydrogen-bond geometry (Å, °)

*D*—H⋯*A*	*D*—H	H⋯*A*	*D*⋯*A*	*D*—H⋯*A*
O2—H2⋯O1^i^	0.82	2.07	2.735 (5)	138
O1—H1⋯N1	0.91 (7)	1.74 (7)	2.569 (5)	151 (6)

Symmetry code: (i) 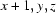.

**Fig. 1.**
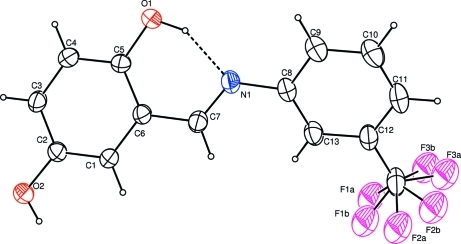
The molecular structure of the title compound, showing the atom-numbering scheme. Displacement ellipsoids are drawn at the 30% probability. Dashed line indicates intramolecular hydrogen bonding.

**Fig. 2.**
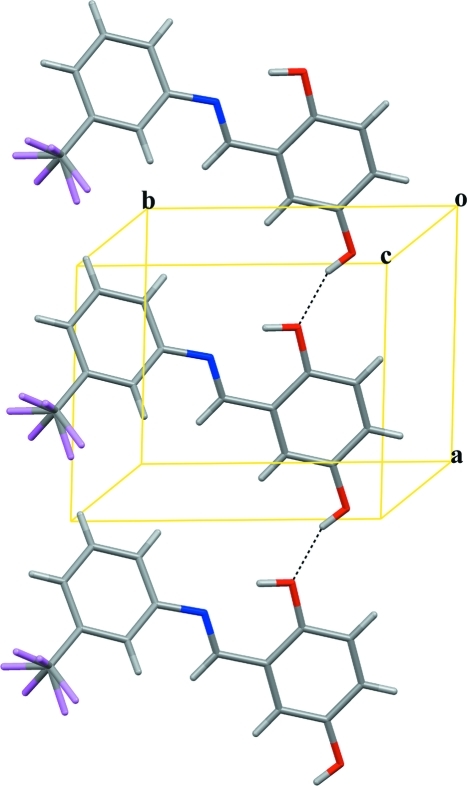
A packing diagram of the title compound; dashed lines indicate intermolecular hydrogen bonds.

## supplementary crystallographic information

### Comment

The present work is part of a structral study of Schiff bases (Gül *et al.*, 2007; Şahin *et al.*, 2009a,b,c) and we report here the structure of (E)-2-[(3- (trifluoromethyl) phenylimino)methyl]-4-hydroxyphenol,(I). The molecular structure of (I) is shown in Figure 1.

Schiff bases often exhibit various biological activities and in many cases were shown to have antibacterial, anticancer, anti-inflammatory and antitoxic properties (Lozier *et al.*, 1975). There are two types of intramolecular hydrogen bonds in Schiff bases, which may be stabilized either in keto-amine (N—H···O hydrogen bond) (Şahin *et al.*, 2005) or phenol-imine (N···H—O hydrogen bond) tautomeric forms (Hadjoudis *et al.*, 1987). The H1 atom in title compound (I) is located on O1 atom, thus the phenol-imine tautomer is favored over the keto-amine form, as indicated by the C5—O1 [1.365 (5) Å], C7—N1 [1.281 (6) Å], C6—C7 [1.448 (6) Å], C5—C6 [1.399 (6) Å] bond lengths. The O1···N1 distance of 2.569 (5)Å is comparable to those observed for analogous hydrogen bond in (*E*)-3-[2-(Trifluoromethyl)phenyliminomethyl]- benzene-1,2-diol [2.568 (3) Å; Temel *et al.*, 2007]. The N1—C7 [1.281 (6) Å] bond length is consistent with significant double-bond character of these bonds. It is known that Schiff bases may exhibit thermochromism or photochromism, depending on the planarity or non-planarity of the molecule, respectively. Therefore, one can expect photochromic properties in (I) caused by non-planarity of the molecules; the dihedral angle the aromatic rings 31.94 (14)°. Molecules are linked into sheets by a combination of O—H···O hydrogen bonds (Table 1). Atom O2 in the asymmetric unit acts as hydrogen-bond donor, *via* H2, connecting this molecule to O1 in a symmetry related molecule at (1 + *x*,*y*,*z*), forming a C(7) chain running parallel to the [100] direction (Fig. 2).

### Experimental

The compound (E)-2-[(3-(trifluoromethyl)phenylimino)methyl]-4-hydroxyphenol was prepared by reflux a mixture of a solution containing 2,5-dihydroxybenzaldehyde (0.0184 g, 0.13 mmol) in 20 ml ethanol and a solution containing 3- trifluoromethylaniline (0.0214 g, 0.13 mmol) in 20 ml ethanol. The reaction mixture was stirred for 1 h under reflux. The crystals of (E)-2-[(3-(trifluoromethyl)phenylimino)methyl]-4-hydroxyphenol suitable for X-ray analysis were obtained from ethylalcohol by slow evaporation (yield % 76; m.p. 404–407 K).

### Refinement

The H1 atom was located in a difference map and refined freely (distances given in Table 1). All other H atoms were placed in calculated positions and constrained to ride on their parents atoms, with C—H=0.93Å and 0.82Å (hydroxyl) and *U*_iso_(H)=1.2*U*_eq_(C) and 1.2*U*_eq_(O). Fluorine atoms are disordered over two alternative positions with refined site occupancies of 0.488 (5) and 0.512 (5).

### Crystal data

**Table:**

C_14_H_10_F_3_NO_2_	*Z* = 2
*M**_r_* = 281.23	*F*(000) = 288
Triclinic, *P*1	*D*_x_ = 1.501 Mg m^−^^3^
Hall symbol: -P 1	Mo *K*α radiation, λ = 0.71073 Å
*a* = 7.1019 (8) Å	Cell parameters from 6675 reflections
*b* = 8.5910 (8) Å	θ = 2.0–27.4°
*c* = 11.0412 (11) Å	µ = 0.13 mm^−^^1^
α = 73.862 (8)°	*T* = 296 K
β = 74.133 (7)°	Plate, brown
γ = 87.431 (8)°	0.49 × 0.32 × 0.02 mm
*V* = 622.10 (11) Å^3^	

### Data collection

**Table:**

Stoe IPDS II diffractometer	2548 independent reflections
Radiation source: fine-focus sealed tube	1490 reflections with *I* > 2σ(*I*)
graphite	*R*_int_ = 0.073
Detector resolution: 6.67 pixels mm^-1^	θ_max_ = 26.5°, θ_min_ = 2.0°
ω scans	*h* = −8→8
Absorption correction: multi-scan (*X-RED32*; Stoe & Cie, 2002)	*k* = −10→10
*T*_min_ = 0.934, *T*_max_ = 0.995	*l* = −13→13
6675 measured reflections	

### Refinement

**Table:**

Refinement on *F*^2^	Primary atom site location: structure-invariant direct methods
Least-squares matrix: full	Secondary atom site location: difference Fourier map
*R*[*F*^2^ > 2σ(*F*^2^)] = 0.100	Hydrogen site location: inferred from neighbouring sites
*wR*(*F*^2^) = 0.293	H atoms treated by a mixture of independent and constrained refinement
*S* = 1.07	*w* = 1/[σ^2^(*F*_o_^2^) + (0.131*P*)^2^ + 0.6957*P*] where *P* = (*F*_o_^2^ + 2*F*_c_^2^)/3
2548 reflections	(Δ/σ)_max_ < 0.001
183 parameters	Δρ_max_ = 0.62 e Å^−^^3^
0 restraints	Δρ_min_ = −0.56 e Å^−^^3^

### Special details

**Table:**

Geometry. All e.s.d.'s (except the e.s.d. in the dihedral angle between two l.s. planes) are estimated using the full covariance matrix. The cell e.s.d.'s are taken into account individually in the estimation of e.s.d.'s in distances, angles and torsion angles; correlations between e.s.d.'s in cell parameters are only used when they are defined by crystal symmetry. An approximate (isotropic) treatment of cell e.s.d.'s is used for estimating e.s.d.'s involving l.s. planes.
Refinement. Refinement of *F*^2^ against ALL reflections. The weighted *R*-factor *wR* and goodness of fit *S* are based on *F*^2^, conventional *R*-factors *R* are based on *F*, with *F* set to zero for negative *F*^2^. The threshold expression of *F*^2^ > σ(*F*^2^) is used only for calculating *R*-factors(gt) *etc*. and is not relevant to the choice of reflections for refinement. *R*-factors based on *F*^2^ are statistically about twice as large as those based on *F*, and *R*- factors based on ALL data will be even larger.

### Fractional atomic coordinates and isotropic or equivalent isotropic displacement parameters (Å^2^)

**Table:**

	*x*	*y*	*z*	*U*_iso_*/*U*_eq_	Occ. (<1)
C1	0.8807 (6)	0.4507 (5)	0.3828 (4)	0.0464 (11)	
H6	0.9747	0.5267	0.3765	0.056*	
C2	0.9124 (6)	0.2884 (5)	0.4264 (4)	0.0463 (11)	
C3	0.7727 (6)	0.1751 (5)	0.4348 (5)	0.0499 (11)	
H3	0.7950	0.0650	0.4627	0.060*	
C4	0.6009 (6)	0.2243 (5)	0.4020 (5)	0.0527 (12)	
H4	0.5075	0.1474	0.4089	0.063*	
C5	0.5675 (6)	0.3880 (5)	0.3588 (4)	0.0447 (10)	
C6	0.7087 (6)	0.5034 (5)	0.3475 (4)	0.0429 (10)	
C7	0.6778 (7)	0.6751 (5)	0.3017 (4)	0.0476 (11)	
H7	0.7704	0.7493	0.3002	0.057*	
C8	0.5029 (7)	0.8960 (5)	0.2113 (5)	0.0510 (11)	
C9	0.3124 (7)	0.9517 (6)	0.2334 (5)	0.0589 (13)	
H9	0.2073	0.8798	0.2822	0.071*	
C10	0.2797 (8)	1.1122 (7)	0.1834 (5)	0.0677 (15)	
H10	0.1525	1.1489	0.1997	0.081*	
C11	0.4334 (8)	1.2195 (6)	0.1095 (5)	0.0650 (15)	
H11	0.4104	1.3281	0.0747	0.078*	
C12	0.6225 (7)	1.1646 (5)	0.0873 (5)	0.0558 (12)	
C13	0.6580 (7)	1.0035 (5)	0.1379 (5)	0.0539 (12)	
H13	0.7856	0.9676	0.1228	0.065*	
C14	0.7854 (9)	1.2793 (6)	0.0089 (6)	0.0711 (16)	
N1	0.5264 (5)	0.7276 (4)	0.2631 (4)	0.0506 (10)	
O1	0.3968 (4)	0.4333 (4)	0.3267 (4)	0.0603 (10)	
H1	0.405 (9)	0.543 (8)	0.297 (6)	0.09 (2)*	
O2	1.0773 (4)	0.2313 (4)	0.4650 (4)	0.0630 (10)	
H2	1.1482	0.3081	0.4575	0.094*	
F1A	0.9366 (14)	1.2094 (11)	−0.0622 (10)	0.1040 (13)	0.488 (5)
F2A	0.8727 (14)	1.3390 (11)	0.0824 (9)	0.1040 (13)	0.488 (5)
F3A	0.7488 (14)	1.4075 (12)	−0.0793 (10)	0.1040 (13)	0.488 (5)
F1B	0.9660 (13)	1.2261 (10)	0.0118 (10)	0.1040 (13)	0.512 (5)
F2B	0.7781 (13)	1.4179 (10)	0.0474 (9)	0.1040 (13)	0.512 (5)
F3B	0.7846 (14)	1.3397 (11)	−0.1146 (10)	0.1040 (13)	0.512 (5)

### Atomic displacement parameters (Å^2^)

**Table:**

	*U*^11^	*U*^22^	*U*^33^	*U*^12^	*U*^13^	*U*^23^
C1	0.042 (2)	0.038 (2)	0.055 (3)	−0.0027 (17)	−0.0144 (19)	−0.0047 (18)
C2	0.038 (2)	0.043 (2)	0.052 (2)	0.0029 (17)	−0.0122 (18)	−0.0050 (19)
C3	0.047 (2)	0.034 (2)	0.062 (3)	0.0019 (17)	−0.012 (2)	−0.0048 (19)
C4	0.045 (2)	0.042 (2)	0.068 (3)	−0.0042 (19)	−0.017 (2)	−0.009 (2)
C5	0.035 (2)	0.042 (2)	0.053 (2)	0.0005 (17)	−0.0104 (18)	−0.0088 (18)
C6	0.039 (2)	0.037 (2)	0.051 (2)	0.0042 (16)	−0.0117 (18)	−0.0098 (17)
C7	0.053 (2)	0.038 (2)	0.050 (2)	0.0008 (18)	−0.015 (2)	−0.0083 (18)
C8	0.055 (3)	0.044 (2)	0.054 (3)	0.013 (2)	−0.021 (2)	−0.009 (2)
C9	0.058 (3)	0.060 (3)	0.058 (3)	0.016 (2)	−0.020 (2)	−0.013 (2)
C10	0.059 (3)	0.070 (3)	0.072 (3)	0.027 (3)	−0.020 (3)	−0.019 (3)
C11	0.080 (4)	0.050 (3)	0.065 (3)	0.029 (3)	−0.026 (3)	−0.015 (2)
C12	0.071 (3)	0.041 (2)	0.057 (3)	0.013 (2)	−0.026 (2)	−0.010 (2)
C13	0.052 (2)	0.046 (2)	0.060 (3)	0.014 (2)	−0.014 (2)	−0.011 (2)
C14	0.084 (4)	0.045 (3)	0.076 (4)	0.015 (3)	−0.025 (3)	−0.001 (3)
N1	0.051 (2)	0.042 (2)	0.056 (2)	0.0084 (16)	−0.0164 (17)	−0.0072 (16)
O1	0.0447 (17)	0.0469 (19)	0.086 (2)	0.0006 (14)	−0.0290 (16)	−0.0024 (17)
O2	0.0441 (17)	0.0491 (19)	0.091 (3)	0.0037 (14)	−0.0281 (17)	−0.0018 (17)
F1A	0.109 (3)	0.078 (3)	0.103 (3)	−0.015 (2)	−0.019 (2)	0.002 (2)
F2A	0.109 (3)	0.078 (3)	0.103 (3)	−0.015 (2)	−0.019 (2)	0.002 (2)
F3A	0.109 (3)	0.078 (3)	0.103 (3)	−0.015 (2)	−0.019 (2)	0.002 (2)
F1B	0.109 (3)	0.078 (3)	0.103 (3)	−0.015 (2)	−0.019 (2)	0.002 (2)
F2B	0.109 (3)	0.078 (3)	0.103 (3)	−0.015 (2)	−0.019 (2)	0.002 (2)
F3B	0.109 (3)	0.078 (3)	0.103 (3)	−0.015 (2)	−0.019 (2)	0.002 (2)

### Geometric parameters (Å, °)

**Table:**

C1—C2	1.374 (6)	C9—C10	1.370 (7)
C1—C6	1.400 (6)	C9—H9	0.9300
C1—H6	0.9300	C10—C11	1.376 (8)
C2—O2	1.378 (5)	C10—H10	0.9300
C2—C3	1.388 (6)	C11—C12	1.383 (7)
C3—C4	1.382 (6)	C11—H11	0.9300
C3—H3	0.9300	C12—C13	1.381 (6)
C4—C5	1.386 (6)	C12—C14	1.463 (8)
C4—H4	0.9300	C13—H13	0.9300
C5—O1	1.365 (5)	C14—F3B	1.319 (11)
C5—C6	1.399 (6)	C14—F3A	1.321 (11)
C6—C7	1.448 (6)	C14—F1B	1.348 (11)
C7—N1	1.281 (6)	C14—F2A	1.357 (12)
C7—H7	0.9300	C14—F2B	1.365 (11)
C8—C13	1.382 (7)	C14—F1A	1.369 (11)
C8—C9	1.395 (6)	O1—H1	0.91 (7)
C8—N1	1.421 (5)	O2—H2	0.8200
			
C2—C1—C6	120.9 (4)	C10—C11—C12	119.4 (4)
C2—C1—H6	119.6	C10—C11—H11	120.3
C6—C1—H6	119.6	C12—C11—H11	120.3
C1—C2—O2	122.8 (4)	C13—C12—C11	120.7 (5)
C1—C2—C3	119.5 (4)	C13—C12—C14	120.1 (4)
O2—C2—C3	117.7 (4)	C11—C12—C14	119.2 (4)
C4—C3—C2	120.6 (4)	C12—C13—C8	119.7 (4)
C4—C3—H3	119.7	C12—C13—H13	120.2
C2—C3—H3	119.7	C8—C13—H13	120.2
C3—C4—C5	120.1 (4)	F3B—C14—F1B	108.3 (7)
C3—C4—H4	120.0	F3A—C14—F1B	124.5 (7)
C5—C4—H4	120.0	F3B—C14—F2A	129.6 (7)
O1—C5—C4	118.9 (4)	F3A—C14—F2A	105.4 (7)
O1—C5—C6	121.2 (4)	F1B—C14—F2A	64.3 (6)
C4—C5—C6	119.9 (4)	F3B—C14—F2B	100.7 (6)
C5—C6—C1	119.0 (4)	F3A—C14—F2B	67.7 (6)
C5—C6—C7	120.9 (4)	F1B—C14—F2B	103.3 (7)
C1—C6—C7	120.1 (4)	F3B—C14—F1A	74.1 (7)
N1—C7—C6	121.8 (4)	F3A—C14—F1A	103.5 (7)
N1—C7—H7	119.1	F2A—C14—F1A	102.9 (7)
C6—C7—H7	119.1	F2B—C14—F1A	131.6 (7)
C13—C8—C9	119.5 (4)	F3B—C14—C12	113.9 (7)
C13—C8—N1	123.1 (4)	F3A—C14—C12	117.6 (6)
C9—C8—N1	117.3 (4)	F1B—C14—C12	116.0 (5)
C10—C9—C8	120.1 (5)	F2A—C14—C12	113.3 (6)
C10—C9—H9	119.9	F2B—C14—C12	113.1 (6)
C8—C9—H9	119.9	F1A—C14—C12	112.7 (6)
C9—C10—C11	120.6 (5)	C7—N1—C8	121.3 (4)
C9—C10—H10	119.7	C5—O1—H1	106 (4)
C11—C10—H10	119.7	C2—O2—H2	109.5
			
C6—C1—C2—O2	−178.4 (4)	C10—C11—C12—C14	−179.8 (6)
C6—C1—C2—C3	0.5 (7)	C11—C12—C13—C8	0.2 (8)
C1—C2—C3—C4	−1.2 (7)	C14—C12—C13—C8	−179.7 (5)
O2—C2—C3—C4	177.7 (4)	C9—C8—C13—C12	−0.2 (7)
C2—C3—C4—C5	0.8 (7)	N1—C8—C13—C12	177.8 (5)
C3—C4—C5—O1	179.8 (4)	C13—C12—C14—F3B	114.4 (7)
C3—C4—C5—C6	0.4 (7)	C11—C12—C14—F3B	−65.5 (8)
O1—C5—C6—C1	179.5 (4)	C13—C12—C14—F3A	152.7 (7)
C4—C5—C6—C1	−1.2 (6)	C11—C12—C14—F3A	−27.2 (10)
O1—C5—C6—C7	0.2 (7)	C13—C12—C14—F1B	−12.3 (10)
C4—C5—C6—C7	179.5 (4)	C11—C12—C14—F1B	167.8 (7)
C2—C1—C6—C5	0.7 (6)	C13—C12—C14—F2A	−83.9 (8)
C2—C1—C6—C7	−180.0 (4)	C11—C12—C14—F2A	96.2 (7)
C5—C6—C7—N1	−3.9 (7)	C13—C12—C14—F2B	−131.4 (7)
C1—C6—C7—N1	176.8 (4)	C11—C12—C14—F2B	48.7 (8)
C13—C8—C9—C10	−0.4 (8)	C13—C12—C14—F1A	32.4 (9)
N1—C8—C9—C10	−178.5 (5)	C11—C12—C14—F1A	−147.5 (7)
C8—C9—C10—C11	1.0 (8)	C6—C7—N1—C8	−176.4 (4)
C9—C10—C11—C12	−0.9 (9)	C13—C8—N1—C7	34.0 (7)
C10—C11—C12—C13	0.3 (8)	C9—C8—N1—C7	−147.9 (5)

### Hydrogen-bond geometry (Å, °)

**Table:**

*D*—H···*A*	*D*—H	H···*A*	*D*···*A*	*D*—H···*A*
O2—H2···O1^i^	0.82	2.07	2.735 (5)	138
O1—H1···N1	0.91 (7)	1.74 (7)	2.569 (5)	151 (6)

Symmetry codes: (i) *x*+1, *y*, *z*.
